# Assessment of cold tolerance in early field emergence in sweet, fiber, and grain sorghums [*Sorghum bicolor* (L.) Moench]

**DOI:** 10.3389/fpls.2026.1842879

**Published:** 2026-09-09

**Authors:** Cristina Patanè, Salvatore L. Cosentino, Giorgio Testa, Valeria Cafaro

**Affiliations:** 1 Consiglio Nazio-nale delle Ricerche (CNR)-Istituto per la BioEconomia (IBE), Sede Secondaria di Catania, Catania, Italy; 2 Dipartimento di Agricoltura, Alimentazione e Ambiente, Università degli Studi di Catania, Catania, Italy

**Keywords:** biomass yield, cold tolerance, early sowing, field emergence, *Sorghum bicolor*

## Abstract

**Introduction:**

Constraints to the adoption of early sowings in biomass sorghum were found in its sensitivity to cold stress during germination.

**Methods:**

A preliminary test was conducted in the laboratory on seven sorghums among sweet, fiber, and grain types, to assess their cold tolerance during germination. Sorghums were germinated at 8 °C and 25 °C (control). At the end of the test, final germination percentage (FG), mean germination time (MGT), and germination index (GI) were calculated. Thereafter, a first-year (2009) field study was conducted under the semi-arid climate of Southern Italy, using 10 cultivars of sorghum, including those from the laboratory test, to assess their response to cold during germination and subsequent seedling emergence. Five sowings were considered: 15 and 30 March (S1 and S2, respectively), 15 and 30 April (S3 and S4, respectively), and 15 May (S5, control). Field emergence was recorded from sowing up to approximately 5 weeks after sowing. At harvest, final plant density, number of leaves per plant, plant height and weight, and biomass yield were measured. A second-year (2010) field experiment was conducted in the same experimental site, using four sorghums among those that performed best in the previous year. Sorghums were sown on 16 March (S1) and 15 May (S5).

**Results:**

FG as assessed in the laboratory was >90% while, at 8 °C, it dropped to 54.9%. At 8 °C, the most tolerant cultivar, in terms of FG, MGT, and GI, was “H128”. Results from the first-year experiment revealed a low seedling emergence (<17%) in all cultivars in S1 (mid-March). Field emergence was positively correlated with maximum air temperature recorded during the 10-day period following sowing, in all cultivars except “Kaoliang” (control). Very low field emergence in early sowing (S1) led to poor plant density in all cultivars except “NK180”, where it matched the predicted value (11 plants m^−2^). Very low temperatures in S1 decreased plant productivity, which was maintained at <8 t ha^−1^ in most cultivars. Biomass yield in S2 (late March) matched that measured in S5 (mid-May). Warmer temperatures (+4–5 °C) in S1 in the second year induced greater field emergence and dry biomass than those measured in the first year for the same sowing times.

**Discussion:**

The availability of cold-tolerant cultivars of sorghum during the early stage of germination and seedling growth may help breeders in their genetic studies for the development of new cultivars that combine cold resilience and high productivity, and help farmers to predict sowing rate based on the expected temperature conditions. In this regard, the sorghum “NK180”, resilient to cold, although low-yielding even in regular sowings, may represent a valuable genetic source for breeding programs to enhance chilling tolerance in the field during the very early stages of growth, among the existing cultivars of sorghum. The cultivar “H128”, also resistant to cold, showed more stable production over sowing times, thus becoming agronomically more suitable. Although seed germination in the laboratory may not be considered a reliable predictor of successful field emergence under very limiting environmental conditions, it can help in a first screening within the available germplasm of sorghum for cold tolerance.

## 1 Introduction

Bioenergy emerges as a promising alternative to conventional fossil fuels, significantly reduces greenhouse gas (GHG) emissions, and mitigates global warming, while diversifying energy sources and ensuring continuous energy supply (Singh et al., 2025). In addition to its environmental benefits, bioenergy contributes to rural development, by creating new job opportunities and promoting agricultural development. Growing dedicated energy crops on marginal lands makes it possible to generate renewable energy without competing with food production, contributing simultaneously to environmental sustainability and agricultural diversification (Osorio-González et al., 2020; Cafaro et al., 2025).

In this context, sorghum [*Sorghum bicolor* (L). Moench] is recognized as a valuable bioenergy crop due to its ability to adapt to different environments (Yu, 2024). As a C4 plant, sorghum has several benefits, including a high rate of photosynthesis, low water needs, and efficient nutrient absorption (Cosentino et al., 2012; Yu, 2024). It can grow on semi-arid or poor-quality soils and requires relatively low amounts of pesticides and fertilizers. Sorghum is well-suited for producing different types of bioenergy. Its grains are rich in starch, its juice contains fermentable sugars, and its biomass has a high cellulose content. All of these can be converted into bioethanol, biogas, and other fuels (Bakari et al., 2023). Additionally, sorghum features a deep root system that helps increase soil organic carbon and reduce fertilizer runoff, improving soil health and providing advantages in sustainable farming systems (Cosentino et al., 2012; Lamb et al., 2022). Sorghum versatility lies both in its agronomic resilience and in its feedstocks diversity. This dual potential makes sorghum well-suited for sustainable bioenergy chains, particularly in areas where water scarcity or soil degradation limits other crops. Moreover, its capacity to maintain acceptable yields under suboptimal conditions reduces production risks and supports its use in marginal areas (Bakari et al., 2023). To highlight the growing relevance of this crop, a bibliographic analysis was carried out using the indexed database Scopus (Donthu et al., 2021). The search string included the terms “*sorghum*” OR “*sorghum bicolor*” AND “*biomass*” OR “*bioenergy*”, resulting in 642 publications across seven European countries (Belgium, France, Germany, Greece, Italy, Portugal, and Spain). Although the number of scientific papers remained relatively low between the mid-1990s and mid-2000s, reflecting the limited role of sorghum in the renewable energy panorama of those years, a clear shift occurred after 2006, coinciding with increasing concerns about energy security, fossil-fuel dependence, and climate change mitigation (International Energy Agency, 2021). Being native to tropical areas, sorghum is highly susceptible to low temperatures during its growth. Studies have been conducted to explore the genetic variability for cold sensitivity within the germplasm of sorghum, aiming at improving its tolerance to low temperatures (Casto et al., 2021). Indeed, in the semi-arid areas of Southern Europe, the adoption of early sowings in March is recommended as a strategy to escape from drought, allowing the crop to benefit from water stored into the soil during the rainy season and save water for irrigation (Patanè et al., 2021). However, its chilling sensitivity especially during the very early stages of the growing season (seed germination and seedling emergence) (Patanè et al., 2008) limits the adoption of early sowings in sorghum, and temperatures often below 10 °C such as those occurring in early spring in Southern Europe delay and reduce plant establishment. This makes early sowing a potentially advantageous but risky practice, strongly dependent on cultivar tolerance to cold. Therefore, identifying cultivars more resilient to cold is crucial to expand the geographic area of crop cultivation.

In the framework of the “BIOSEA” project funded by the Italian Ministry of Agriculture and Forestry, a study was conducted both in the laboratory at low temperature (8 °C) and under field conditions, during early sowings in March. The aims of this research were (i) to determine whether genetic variability for cold tolerance exists within a pool of sorghum cultivars during seed germination and seedling emergence; (ii) to identify those more resilient to cold; and (iii) to investigate the limits imposed by low temperatures on the adoption of early sowings in semi-arid areas of Southern Europe.

## 2 Materials and methods

### 2.1 Germination test

A preliminary test was conducted in the laboratory for seed germination assessment in sorghum. Seven cultivars were considered (**Table 1**).

**Table 1:** List of the cultivars of sorghum assessed for germination in laboratory.

Cultivar	Type	Seed company
Brandes	Sweet	U.S. Sugar Crops Field Station
LP35	Sweet	A.Biotec (Italy)
MN1500	Sweet	Mississippi Agricultural Experiment Station (USA)
Keller	Sweet	Mississippi Agricultural Experiment Station (USA)
Wray	Sweet	Mississippi Agricultural Experiment Station (USA)
H128	Fiber	Protosemences (France)
Kaoliang	Grain (control)	USDA (USA)

Seeds were germinated at a constant temperature of 8 °C (suboptimal) and 25 °C (optimal, control) (Patanè et al., 2016), in a thermostatically controlled (± 1 °C) incubator in the dark. For each cultivar and temperature, 50 seeds, four times replicated, were placed in 9-cm Petri dishes, on a single filter paper moistened with 7 mL of distilled water. Dishes were sealed with Parafilm to prevent water loss by evaporation. Seed germination was scored daily, on seeds whose radicle was at least 2 mm long. The test was stopped after 96 h in the absence of any observed germination.

### 2.2 Two-year field experiment

The experiment was conducted in an open field during the seasons 2009–2010 in a hilly site of Sicily (South Italy, 500 m a.s.l., 37°27′ N latitude, 14°14′ E longitude) on a typical Vertic Xerofluvent (**Table 2**).

**Table 2:** Soil physicochemical characteristics of the site of field experiment.

Soil characteristics	Unit	Value
Sand	%	64.0
Loam	%	23.5
Clay	%	12.5
Organic matter	%	1.51
pH	–	8.3
Total N	‰	1.01
Available P_2_O_5_	ppm	35.1
Exchangeable K_2_O	ppm	403.1
Bulk density	g cm^−3^	1.2
Field capacity (−0.03 MPa)	%	21.0
Wilting point (−1.5 MPa)	%	11.0

#### 2.2.1 First-year experiment

##### 2.2.1.1 Study design

In the first year, 10 cultivars of sorghum [*Sorghum bicolor* (L.) Moench] among distinct types (fiber, sweet, and grain) were assessed (**Table 3**). Seven of them (“Brandes”, “LP35”, “MN1500”, “Keller”, “Wray”, “H128”, and “Kaoliang”) were common to the laboratory test for seed germination assessment, as previously described (see Section 2.1). The cv. “Kaoliang” was adopted as a control, as it has been reported as tolerant to cold (Franks et al., 2006). At the time of the experiment, the seeds were less than 1 year old, kept at room temperature (10–20 °C) before the start of the field experiment.

**Table 3:** List of the cultivars of sorghum assessed in the first-year field experiment.

Cultivar	Type	Seed company
Brandes	Sweet	U.S. Sugar Crops Field Station
LP35	Sweet	A.Biotec (Italy)
MN1500	Sweet	Mississippi Agricultural Experiment Station (USA)
Keller	Sweet	Mississippi Agricultural Experiment Station (USA)
Wray	Sweet	Mississippi Agricultural Experiment Station (USA)
Soave	Sweet	Società Italiana Sementi (Italy)
H128	Fiber	Protosemences (France)
Roce	Sweet	Società Italiana Sementi (Italy)
NK180	Grain	Northrup King (USA)
Kaoliang	Grain (control)	USDA (USA)

In order to evaluate the cold tolerance of the different cultivars during early field emergence, five sowing times were considered: 15 March (S1), 30 March (S2), 15 April (S3), 30 April (S4), and 15 May (S5, control). S1, S2, and S3 were considered as early sowings for sorghum in semi-arid regions of the Mediterranean area, based on the thermal requirements of the species to germinate (Patanè et al., 2008). A split-plot design in an RCBD layout with three blocks (replicates) was adopted in the field, assigning “sowing time” to the main plot and “cultivar” to the sub-plot. A single plot measured 8.4 m^2^ (four rows spaced 0.70 m).

##### 2.2.1.2 Agronomic management

Before sowing, 60 and 100 kg ha^−1^ of N (as ammonium sulfate) and P_2_O_5_ (as mineral superphosphate), respectively, were applied in the field. A month after plant emergence, 60 kg ha^−1^ of N (as ammonium nitrate) was distributed as top dressing.

At sowing, three seeds in holes 0.13 m distant were used. Plant thinning was not required, because in most cases, seedling density was even below the predicted final plant density (11 plants m^−2^).

During the growing season, irrigation was applied by a drip irrigation system, to meet crop requirements (100% evapotranspiration restoration). A total of 416.7 mm of water in S1 and S2, 511.9 mm in S3, 559.5 mm in S4, and 567.5 mm in S5 were distributed.

Plants were harvested between September and November, according to sowing time and cultivar.

##### 2.2.1.3 Data collection

Throughout the plant emergence period (from March to June), air and soil maximum and minimum temperatures were measured using a weather station connected to a data logger (CR10, Campbell Scientific, Inc. Logan, UT, USA).

**Figure 1:**
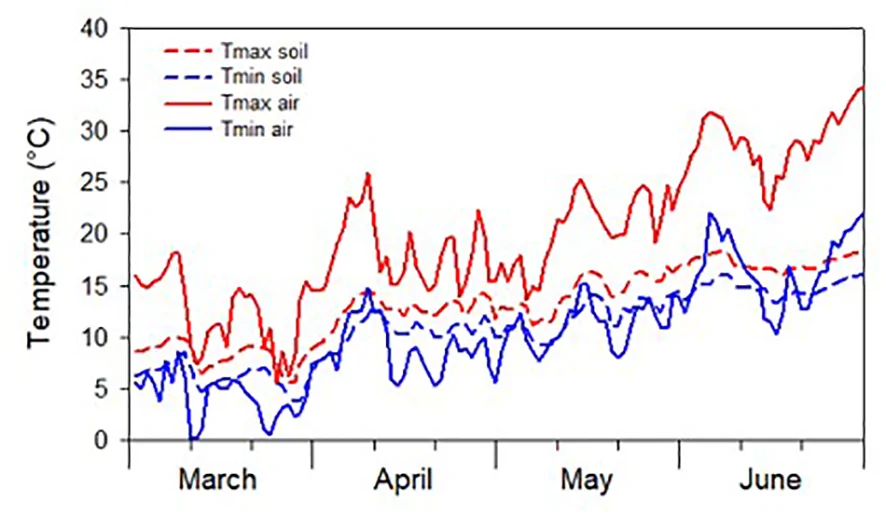
Course of daily air and soil temperatures (maximum and minimum) recorded during seed germination and following seedling emergence in open field (year: 2009).

The course of air and soil temperatures in their maximum and minimum values was recorded during the first stages of crop growth in the field. Seeds of the first sowing (S1, mid-March) experienced soil temperatures (both minimum and maximum) below 10 °C until the end of March (**Figure 1**).

Soil temperature rapidly rose from April onwards, exceeding 15 °C (maximum temperature) in mid-May, when the last sowing (S5) was made, and peaking at 32 °C in early June. Overall, wider fluctuations were observed in air temperatures. In March, temperature rapidly dropped after the first 10-day period to 8 °C (*T*_max_) and 1 °C (*T*_min_) recorded in the last decade of the month. Seeds of S2 (sowing of late March) benefited from increasing air temperatures for their germination (*T*_max_ up to 26 °C and *T*_min_ up to 15 °C), but during the second decade of April, temperatures again dropped, in both maximum and minimum values. Air thermal conditions became favorable again for seed germination in mid-May sowing (S5).

Maximum and minimum air temperatures were also measured in the second year during seed germination and subsequent seedling emergence (from March to June). Overall, thermal course was warmer in the second season. Throughout the early steps of germination in the field (i.e., approximately 10 days after sowing), in 2010, seeds of the first sowing (that in mid-March) experienced temperatures that were up to 4–5 °C higher than those measured in 2009 (**Table 4**). Thermal differences became less evident with the second sowing (that in mid-May), when *T*_max_ of the last 10-day period in May (23.02 °C in 2009 and 25.27 °C in 2010) matched the optimum for seed germination of sorghum (~23–25 °C). During the plant growth period, the number of emerged seedlings (with the first true leaf and the second not fully expanded) was observed once a week in all holes of the two central rows, until no further seedling emergence occurred (approximately 5 weeks after sowing).

**Table 4:** Maximum and minimum air temperature (mean of the 10-day period) recorded from March to June in the 2 years of field experiment.

Month	Decade	2009		2010	
		*T*_max_ (°C)	*T*_min_ (°C)	*T*_max_ (°C)	*T*_min_ (°C)
March	1	15.79	5.86	13.06	4.36
	2	11.01	4.17	14.32	5.86
	3	10.70	3.76	16.22	7.11
April	1	20.12	10.97	16.36	7.06
	2	16.35	8.17	16.93	8.44
	3	17.87	8.95	20.62	9.88
May	1	17.38	10.16	21.52	11.62
	2	22.24	12.41	23.98	13.63
	3	23.02	12.82	25.27	14.42
June	1	29.61	18.58	26.85	15.15
	2	27.17	15.02	27.13	15.97
	3	30.49	18.58	30.79	16.99

The number of leaves per plant was measured weekly on 8 labeled plants of the two central rows, starting when the second leaf was fully expanded until the last leaf was emitted.

At harvest, plant height was measured on a sample of 10 plants for each cultivar and sowing time, randomly selected on the two central rows (five plants per row), then all cultivars were evaluated for productivity, separately per sowing time. To this end, total aboveground fresh biomass was measured in all plants of the two central rows, after removing the border plants. These plants were also counted for actual final plant density calculation. Dry matter was determined on plant samples oven-dried at 70 °C until constant weight, for total dry biomass yield estimation. The dry weight of single plants was also measured in a sample of 10 plants randomly selected among those collected for yield estimation.

#### 2.2.2 Second-year experiment

A second-year (2010) field experiment was conducted in the same experimental site, adopting four sorghums among those that performed best in the previous year for productivity (“Brandes” and “Keller”) or cold tolerance during emergence in field (“LP35” and “NK180”). Two sowing times were considered: 16 March (S1) and 15 May (S5, control). The aim of the second-year open-field experiment was to verify the performance of those cultivars of sorghum, which better behaved in the first-year experiment under the extreme sowing times. Air temperature (maximum and minimum) was measured during the plant emergence period (from March to June), using the same weather station previously described. The same agronomical practices, from sowing to harvest, described for the field experiment in 2009 (see Section 2.2.1) were applied in the field. A total of 422.5 and 571.5 mm of irrigation water were distributed in S1 and S5, respectively. Plants were harvested between September and November, and at harvest, total aboveground dry biomass was measured.

### 2.3 Calculations and data analysis

#### 2.3.1 Seed germination experiment

At the end of the germination test, final germination (FG, %) and mean germination time (MGT, days) were calculated, according to Toscano et al. (2025), and the germination index (GI, unitless), which is a combined measure of speed and completeness of germination, was calculated, according to Toscano et al. (2017).

The course of germination with time, for each cultivar at each temperature, was described by a nonlinear iterative regression (SIGMAPLOT^®^ 11.0 software, Systat Software Inc., San Jose, CA, USA), using the sigmoidal logistic model with three parameters (Toscano et al., 2025):


$$y=\;\frac{a}{1+{\left(\frac{x}{x0}\right)}^{b}}$$

where *a* is the maximum value of *y* (i.e., maximum germination), *x* is the time (days) after seed imbibition, *x*_0_ is the time (days) to reach 50% of maximal germination, and *b* is a fitting parameter of the curve (Patanè et al., 2016).

Data on final percentage germination, previously arcsine transformed, and those of MGT, were statistically analyzed by a completely randomized two-way analysis of variance (ANOVA) using Minitab Statistical Software version 19, LLC, considering *temperature*, *cultivar*, and their interaction as sources of variation. Before ANOVA, the normality of residuals was checked by means of the Shapiro–Wilk test, and the homoscedasticity was tested by means of Bartlett’s test. The independence of data was assumed by random samplings. Means were separated by Tukey’s test at 95% confidence level.

Data on GI were heteroscedastic, according to the Bartlett’s test; therefore, they were statistically analyzed separately at 8 °C and 25 °C (one-way ANOVA) using Minitab Statistical Software version 19, LLC, considering *cultivar* as sources of variation.

#### 2.3.2 First-year field experiment

Data on field emergence, plant density, plant height and weight, number of leaves, and final dry biomass yield measured in the first year of field experiment were statistically analyzed by a two-way (*sowing time* × *cultivar*) ANOVA, using Minitab Statistical Software version 19, LLC.

A Pearson’s correlation test was conducted between values of final plant emergence measured for each sowing time and those of air and soil temperatures (both maximum and minimum) recorded in the first 10-day period after sowing.

The number of leaves measured in the first year throughout plant growth was regressed against thermal time up to last leaf emission, separately per cultivar. Thermal time [or Growing Degree Days (GDD)] was calculated according to the formula (Patanè et al., 2019):


$$GDD\;({}^{\circ}Cd)=\;\overset{n}{\underset{i=1}{\sum }}(\frac{H_{i}+L_{i}}{2}-T_{b})$$

where *n* is the number of days up to last leaf emission; *H_i_* and *L_i_* are maximum and minimum air temperature (°C), respectively, of day *i*; and *T*_b_ = base temperature (assumed as 10 °C for the growth of sorghum, Anda and Pintér, 1994) (if *L_i_*< *T*_b_, then *L_i_* = *T*_b_, Patanè et al., 2019). The *b* coefficient of the regression represents the rate of leaf emission (number of leaves per day, *n d*).

The relationship of dry biomass vs. plant density, separately per group of sorghums (that of sweet and fiber types and that of grain types), was described using the following two-parameter exponential model:


$$y=a(1-e^{-bx})$$

where *y* is dry biomass, *a* is the maximum value of *y* (i.e., maximum dry biomass), *x* is plant density, and *b* is a fitting parameter of the curve.

#### 2.3.3 Second-year field experiment

Data on field emergence and final dry biomass measured in the second**-**year experiment were statistically analyzed together with those of the first**-**year experiment by a three-way (*year* × *sowing time* × *cultivar*) ANOVA, using Minitab Statistical Software version 19, LLC. Cultivars (“Brandes”, “LP35”, “Keller”, and “NK180”) and sowing times (S1, mid-March, S5, mid-May) common to the 2 years of experiment were considered. Means were separated by Tukey’s test at 95% confidence level.

The relationship of plant emergence in field vs. air *T*_max_ of the 10-day period following sowing, considering both years of experiment, was studied.

## 3 Results

### 3.1 Seed germination in laboratory

**Figure 2:**
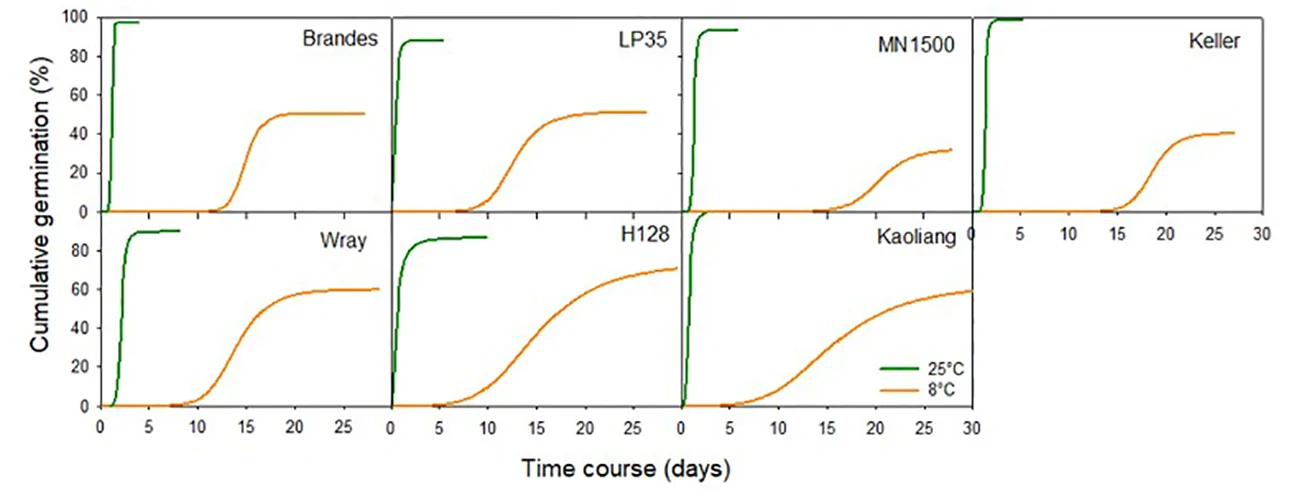
Time course of cumulative germination at 25 °C and 8 °C in seven cultivars of sorghum, as described by the three-parameter sigmoidal model.

**Figure 2** illustrates the time course of cumulative seed germination in seven cultivars of sorghum at 25 °C and 8 °C. The sigmoidal pattern indicates that germination proceeded slowly in its initial steps, and increased afterwards up to a maximum. The initial phase of low germination was overall negligible at optimal temperature (25 °C) and was lengthened at suboptimal temperature (8 °C), especially in “MN1500” and “Keller”.

Across cultivars, final germination at 25 °C exceeded 90%, while at 8 °C, it significantly dropped to 54.87% (**Table 5**). However, an interactive effect “*temperature* × *cultivar*” was highlighted by ANOVA. Indeed, no differences were detected at optimal temperature among cultivars, but they behaved differently at 8 °C: the highest FG (70%) corresponded to “H128”, whereas the lowest FG corresponded to “Keller” (33.33%) and “MN1500” (25.00%).

**Table 5:** Effects of cultivar (*C*), temperature (*T*), and their interaction on final seed germination (FG) and mean germination time (MGT) in sorghum (laboratory experiment).

Cultivar	FG (%)			MGT (d)		
	25 °C	8 °C	Average (*T*)	25 °C	8 °C	Average ± sd (*T*)
Brandes	97.00 a	52.10 c	74.55 ± 8.6 AB	1.81 fg	18.18 b	9.99 ± 3.10 B
LP35	89.00 a	53.00 bc	71.00 ± 7.8 AB	2.10 f	18.25 b	10.18 ± 3.05 B
MN1500	93.01 a	25.00 d	59.00 ± 13.0 C	1.90 fg	20.99 a	11.45 ± 3.60 A
Keller	98.32 a	33.33 d	65.83 ± 12.3 BC	1.86 fg	21.70 a	11.78 ± 3.77 A
Wray	89.33 a	65.33 bc	77.33 ± 5.7 A	1.62 fg	17.00 c	9.31 ± 2.90 C
H128	88.92 a	70.00 b	79.46 ± 3.9 A	1.52 fg	15.00 e	8.26 ± 2.55 D
Kaoliang	100.00 a	61.32 bc	80.66 ± 7.4 A	1.24 g	16.03 d	8.64 ± 2.80 D
Average ± sd (*C*)	93.65 ± 4.76 A	51.44 ± 16.66 B		1.72 ± 0.28 B	18.16 ± 2.46 A	
CV (%)	5.09	32.38		16.51	13.56	

Values followed by the same letter (uppercase letters for the main effects and lowercase letters for the interaction effect) are not significantly different at *p* ≤ 0.05 (Tukey’s test).

Germination speed (as MGT), as well, was significantly affected by “*cultivar*” and “*temperature*”. Across cultivars, under favorable thermal conditions, seeds germinated in 1.72 days (MGT) but at 8 °C germination was significantly lengthened, and seeds required almost 18 days to germinate. Across temperatures, the fastest in germination were “H128” and “Kaoliang”, whereas the slowest were “MN1500” and “Keller”. As for FG, the two experimental factors significantly interacted on MGT. Indeed, all cultivars except “Kaoliang” did not differ in germination speed at 25 °C, but under cool conditions, “H128” was much faster, taking 15 days (MGT) to germinate.

GI, which reflects both speed and final germination performance (the higher is the GI, the faster and the higher is the germination) (Toscano et al., 2017), was significantly reduced at 8 °C (0.88 vs. 14.5 at 25 °C). Among cultivars, at 25 °C, GI was the highest in “Kaoliang” (22.00) and the lowest in “MN1500” (7.50). When the temperature was lowered to 8 °C, the highest GI (1.41) was recorded for “H128”. Conversely, a low GI value (0.30) was calculated for “Keller”, reflecting both reduced final germination percentage and the extended time required by seeds to germinate at this temperature (**Table 6**).

**Table 6:** Effects of cultivar on the germination index (GI) in sorghum at 25 °C and 8 °C (laboratory experiment).

Cultivar	GI	
	25 °C	8 °C
Brandes	14.50 c	0.89 b
LP35	9.98 d	0.90 b
MN1500	7.50 e	0.79 b
Keller	14.00 c	0.30 c
Wray	15.33 c	0.92 b
H128	18.47 b	1.41 a
Kaoliang	22.00 a	0.98 b
Average ± sd	14.5 ± 4.87	0.88 ± 0.33
CV (%)	33.50	36.86

Values followed by the same letter are not significantly different at *p* ≤ 0.05 (Tukey’s test).

### 3.2 Field experiment (first year)

#### 3.2.1 Field emergence

Field emergence of sorghum was significantly affected by both the experimental factors (*S*, *C*, *p*< 0.001) (**Table 7**). It was the lowest in the earliest sowing time (S1, 7.32%). Unexpectedly, the emergence in late March (S2) matched that in mid-May (S5, control), where the percentage of seedling emerged across cultivars approached 50%.

**Table 7:** Effects of sowing time and cultivar on field emergence and some productivity traits in sorghum.

Main effect		Field emergence (%)	Plant density (p m^−2^)	Plant height (cm)	Leaves (n)	Plant weight (g DW)	Dry biomass (t ha^−1^)
Sowing time (*S*)	S1 (16 March)	7.32 ± 1.34 d	3.31 ± 0.60 d	174.7 ± 7.82 d	15.6 ± 0.62 a	261.0 ± 19.8 a	7.62 ± 1.18 c
	S2 (30 March)	46.96 ± 2.91 a	10.01 ± 0.49 a	184.1 ± 8.44 cd	16.1 ± 0.18 a	197.6 ± 12.9 b	16.39 ± 1.00 a
	S3 (16 April)	33.12 ± 2.57 b	7.80 ± 0.66 b	193.8 ± 9.88 bc	14.5 ± 0.21 b	192.4 ± 14.5 b	13.36 ± 0.98 b
	S4 (30 April)	22.68 ± 3.21 c	5.02 ± 0.59 c	203.0 ± 10.1 ab	14.4 ± 0.32 b	252.4 ± 22.4 a	11.01 ± 1.02 b
	S5 (15 May)	45.48 ± 2.99 a	10.60 ± 0.76 a	212.0 ± 8.81 a	14.0 ± 0.34 b	185.7 ± 11.8 b	18.02 ± 1.12 a
Cultivar (*C*)	Brandes	27.68 ± 4.36 cd	10.21 ± 1.08 b	190.1 ± 7.66 d	14.3 ± 0.52 b	213.0 ± 23.7 cd	18.73 ± 0.99 a
	LP35	38.42 ± 6.06 b	6.51 ± 1.24 cd	201.8 ± 5.40 b-d	14.7 ± 0.56 b	213.3 ± 13.4 cd	12.91 ± 2.17 cd
	MN1500	21.65 ± 2.91 d	7.73 ± 0.68 c	212.3 ± 4.05 bc	14.9 ± 0.31 b	239.7 ± 11.9 bc	15.47 ± 1.31 ab
	Keller	21.74 ± 4.11 d	4.98 ± 0.90 d	237.1 ± 10.4 a	15.2 ± 0.89 b	286.5 ± 26.7 ab	13.40 ± 2.15 bc
	Wray	18.41 ± 2.64 d	6.59 ± 0.88 cd	243.5 ± 6.95 a	16.9 ± 0.38 a	315.5 ± 24.5 a	19.15 ± 1.75 a
	Soave	36.38 ± 5.40 bc	8.49 ± 1.13 bc	216.9 ± 6.67 b	15.0 ± 0.40 b	171.6 ± 13.7 d	14.35 ± 1.48 bc
	H128	37.09 ± 5.26 b	7.08 ± 0.75 cd	195.6 ± 5.31 cd	13.9 ± 0.38 b	211.8 ± 15.3 cd	13.47 ± 0.80 bc
	Roce	34.93 ± 6.04 bc	6.38 ± 1.02 cd	164.6 ± 4.25 e	15.2 ± 0.62 b	215.1 ± 28.0 cd	11.18 ± 1.61 cd
	NK180	54.28 ± 5.71 a	12.97 ± 0.87 a	78.3 ± 3.05 f	15.0 ± 0.40 b	71.2 ± 4.1 e	7.92 ± 0.60 de
	Kaoliang	18.55 ± 2.58 d	2.54 ± 0.50 e	195.2 ± 5.94 cd	14.7 ± 0.62 b	240.4 ± 17.3 bc	6.20 ± 0.96 e
Significance	*S*	***	***	***	***	***	***
	*C*	***	***	***	***	***	***
	*S* × *C*	***	*	***	***	***	***

For the main effects, values (mean ± SE) followed by the same letter are not significantly different at *p*< 0.05 (Tukey’s test) (year: 2009).

DW: dry weight; significant at *p* ≤ 0.05 (*) and *p* ≤ 0.001 (***).

**Figure 3:**
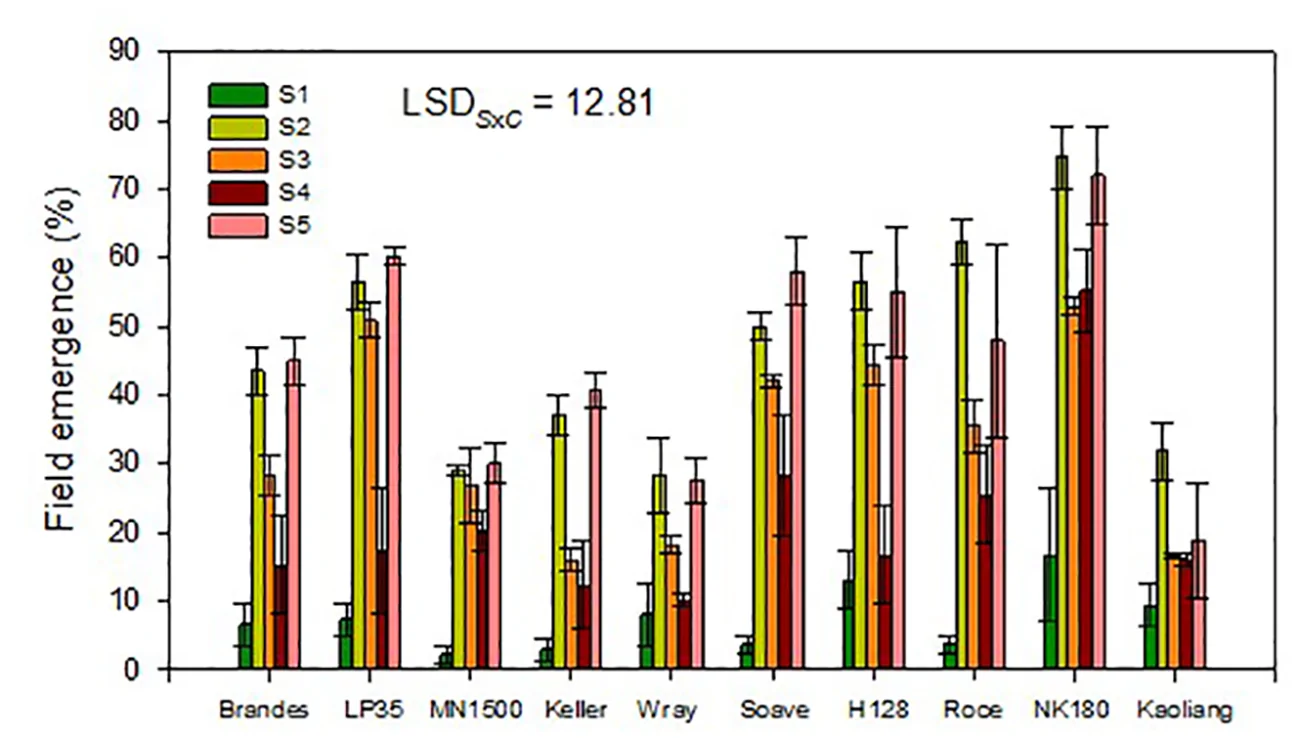
Field emergence in sorghum in relation to sowing time (S1: 16 March; S2: 30 March; S3: 16 April; S4: 30 April; S5: 15 May) and cultivar (year: 2009). Small vertical bars indicate the standard error (*n* = 3).

Among cultivars, the best performing was “NK180”, which was the only sorghum whose field emergence, across sowing times, exceeded 50%. Very low field emergence (<20%) occurred in “Wray” and “Kaoliang”. A significant interaction “*S* × *C*” (*p*< 0.001) was highlighted by ANOVA (**Figure 3**). Indeed, when excluding the first sowing (that of mid-March), when plants emerged in a very low rate in all cultivars, field emergence differed with cultivar and sowing time, keeping constant with sowings from April onwards in some cultivars (e.g., “MN1500”), or fluctuating in some other (e.g., “Brandes”, “LP35”, and “Soave”). Wide genetic differences were observed in the sowing of late April, when emergence percentages ranging between 10.15% (“Wray”) and 55.22% (“NK180”) were measured.

The strict correlation (*r* = 0.94**), found between final germination measured in laboratory at 8 °C for the seven assessed cultivars of sorghum and plant emergence measured in field in the earliest sowing (S1, mid-March), revealed how those cultivars possessing high cold tolerance during germination in the laboratory performed better during germination and following seedling emergence in the field under low temperatures occurring with early sowing.

#### 3.2.2 Relationship of plant emergence vs. temperature

Data of field emergence measured in each cultivar of sorghum were plotted against the average of soil and air temperatures (minimum and maximum) recorded during the 10-day period following sowing. Significant correlations with soil temperatures were found only in “MN1500”, “Soave”, and “NK180” (**Table 8**). Conversely, plant emergence was positively correlated with maximum air temperature in all cultivars except “Kaoliang”. The slope value (*b* coefficient) of these linear relationships provided information on the magnitude of the emergence response to temperatures in each cultivar. Among sorghums, “LP35”, “Soave”, and “NK180” seemed to be more sensitive to thermal increases (or decreases) during seed germination, having higher (or lower) seedling emergence rates (*b*-value) for each degree of thermal variation. Conversely, “Kaoliang”, having low values of the *b* coefficient, can be considered the cultivar most tolerant to thermal changes (and thus thermal decrease) in terms of plant emergence.

**Table 8:** Values of Pearson correlation parameter *r* (correlation coefficient) and *b* coefficient (emergence rate) of the regression between temperature (max and min of soil and air) as the average of the 10-day period following sowing, and plant emergence in the field, in 10 cultivars of sorghum.

Cultivar	Soil *T*_max_		Soil *T*_min_		Air *T*_max_		Air *T*_min_	
	*r*	*b* (% °C^−1^)	*r*	*b* (% °C^−1^)	*r*	*b* (% °C^−1^)	*r*	*b* (% °C^−1^)
Brandes	0.75^ns^	4.96	0.71^ns^	4.92	0.92**	3.91	0.79^ns^	4.47
LP35	0.75^ns^	7.18	0.74^ns^	7.30	0.85*	5.21	0.71^ns^	5.80
MN1500	0.87*	3.92	0.86*	4.01	0.92**	2.67	0.91*	3.50
Keller	0.73^ns^	4.69	0.69^ns^	4.59	0.93**	3.83	0.82*	4.45
Wray	0.67^ns^	2.46	0.63^ns^	2.42	0.87*	2.07	0.72^ns^	2.28
Soave	0.89*	7.40	0.87*	7.52	0.96**	5.17	0.89*	6.35
H128	0.68^ns^	5.52	0.65^ns^	5.53	0.83*	4.35	0.67^ns^	4.71
Roce	0.66^ns^	5.76	0.62^ns^	5.66	0.88*	4.93	0.83*	6.17
NK180	0.82*	7.42	0.79^ns^	7.42	0.96**	5.58	0.96**	7.46
Kaoliang	0.35^ns^	1.12	0.30^ns^	1.00	0.67^ns^	1.39	0.66^ns^	1.82

Significant at *p* ≤ 0.05 (*) and *p* ≤ 0.01 (**); not significant (ns).

Given the significant correlation found with maximum air temperatures in most cultivars, values of the *x*-axis intercept of the regression of plant emergence vs. maximum air temperatures (*T*_max_), as the average of the 10-day period after sowing, were assumed as an estimation of the theoretical minimum or base thermal threshold for plant emergence. Base *T*_max_ > 10 °C estimated in “Brandes”, “Keller”, “Soave”, and “Roce” (**Table 9**) indicated higher thermal requirements in these cultivars for seed germination in the field. Other cultivars (“H128” and “NK180”) appeared less sensitive to cold during the very early stages of crop growth, having a base *T*_max_ for seedling emergence of 7.74 °C and 7.54 °C, respectively. As expected, the most tolerant was “Kaoliang” (*T*_max_ = 3.94 °C).

**Table 9:** Values of theoretical minimum or base air *T*_max_ (*x*-intercept) for plant emergence in the field in the 10 cultivars of sorghum.

Cultivar	*x*-intercept (°C)
Brandes	10.18
LP35	9.88
MN1500	9.16
Keller	11.58
Wray	8.35
Soave	10.22
H128	7.74
Roce	10.19
NK180	7.54
Kaoliang	3.94

#### 3.2.3 Plant density at harvest

**Figure 4:**
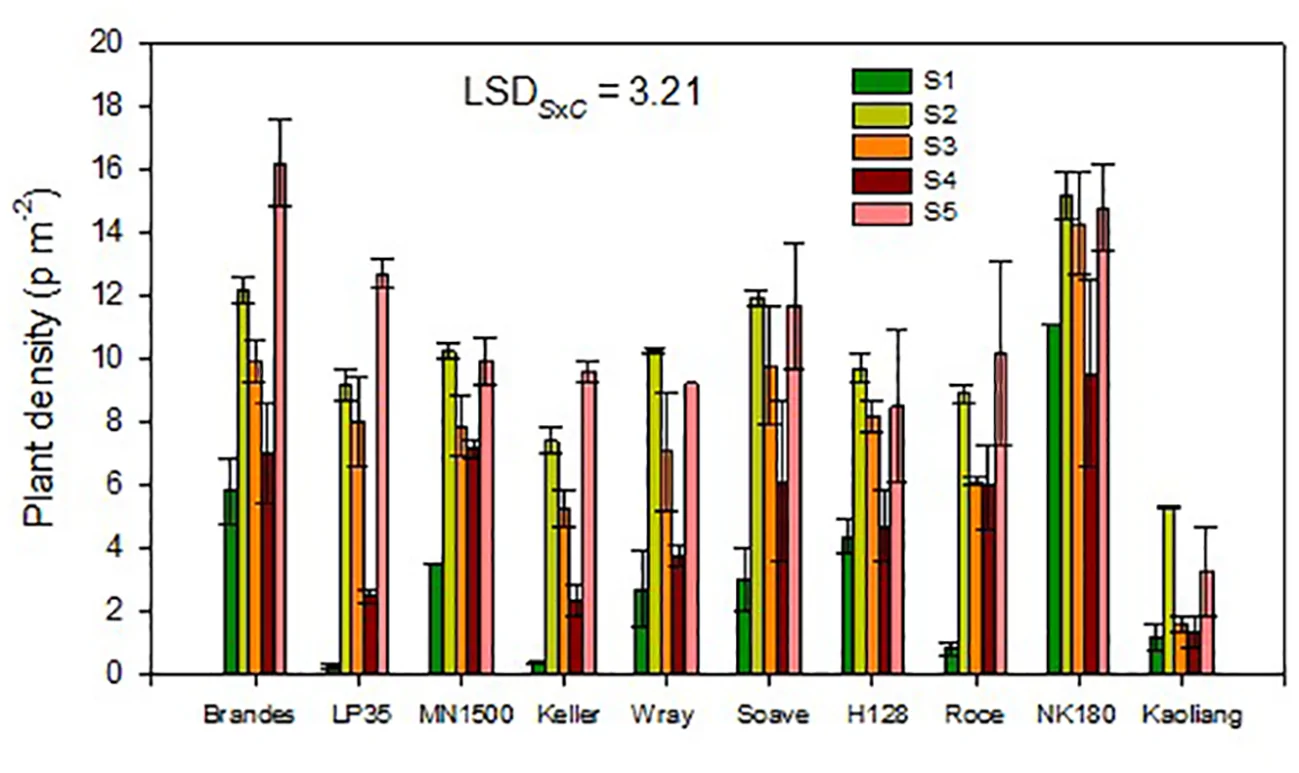
Plant density at harvest in sorghum in relation to sowing time (S1: 16 March; S2: 30 March; S3: 16 April; S4: 30 April; S5: 15 May) and cultivar (year: 2009). Small vertical bars indicate the standard error (*n* = 3).

According to field emergence, final plant density was significantly affected by sowing time and cultivar (*p<* 0.001). Plant population matched the expected value (~11 plants m^−2^) only with sowings in late March (S2) and mid-May (S5). Very low field emergence with early sowing in mid-March led to poor plant density (<3.5 plants m^−2^). Among cultivars, the highest (12.97 plants m^−2^) and the lowest (2.54 plants m^−2^) densities were measured in “NK180” and “Kaoliang”, respectively. Low plant population (<5 plants m^−2^) was also recorded in “Keller”. As for field emergence, significant “*S* × *C*” was found at ANOVA. Indeed, while in some cultivars plant population was low (≤5 p m^−2^) at all sowing times (e.g., Kaoliang), other sorghums (e.g., “MN1500” and “Soave”) achieved a plant density that did not change much with the time of sowing (when excluding that very early in mid-March) (**Figure 4**). High plant density (≥10 p m^−2^) was measured in cultivar “NK180”, irrespective of sowing time.

**Figure 5:**
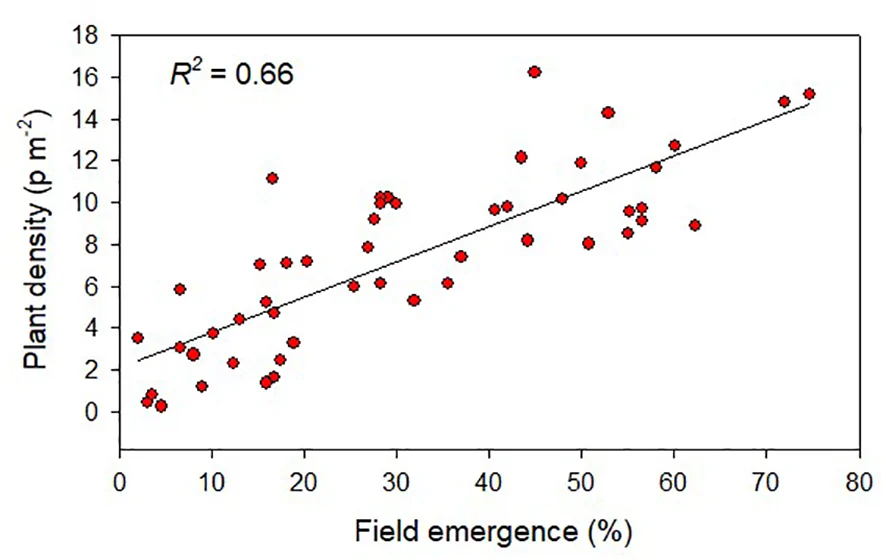
Relationship of plant emergence in the field and final plant density in sorghum.

When the pool of data of all sowing times and cultivars was considered, a strict linear relationship (*R*^2^ = 0.66) was found between field emergence and plant density (**Figure 5**).

#### 3.2.4 Plant height and number of leaves per plant at harvest

Plant height and number of leaves per plant were measured at harvest. Plant growth was affected by sowing time, and plants significantly taller were observed in the last sowing (S5, mid-May). Conversely, across cultivars, plants were smaller in the earliest sowing (S1, mid-March). Plant height also changed with cultivar, and across sowing times, the tallest plants were those of “Wray” and “Keller” (>230 cm), both sweet types. As expected, plants significantly smaller were measured in “NK180” (<80 cm), as grain type.

**Figure 6:**
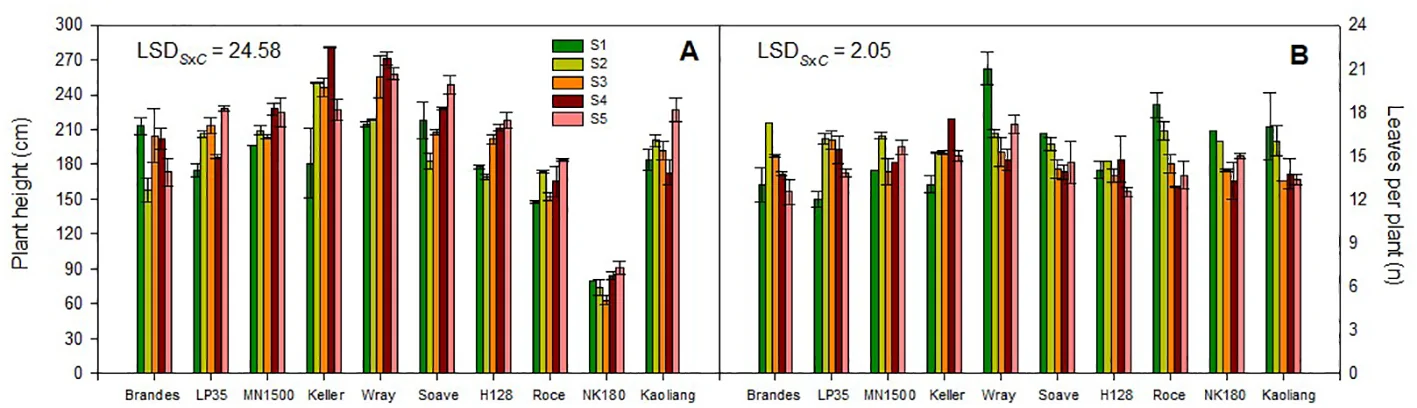
Plant height **(A)** and number of leaves per plant **(B)** at harvest in sorghum in relation to sowing time (S1: 16 March; S2: 30 March; S3: 16 April; S4: 30 April; S5: 15 May) and cultivar (year: 2009). Small vertical bars indicate the standard error (*n* = 3).

A significant “*S* × *C*” was found at ANOVA also for plant height. Some cultivars (e.g., “MN1500”, “Roce”, and “NK180”) achieved a final height similar at all sowing times. Other cultivars (e.g., “LP35” and “Keller”) were smaller in S1 (that of mid-March) or in S2 (that of late March) (e.g., “Brandes” and “Soave”) or in both sowings (e.g., “Wray” and “H128”) (**Figure 6A**).

The final number of leaves per plant significantly varied with sowing time and cultivar. Plants of S1 and S2 (both in March), although smaller, had more leaves (≥16). The same final number of leaves per plant was measured in all cultivars, except in “Wray”, where more leaves per plant than in the other sorghums were counted. The two experimental factors significantly interacted on this trait (*p*< 0.001). In fact, while minor variations with the time of sowing were observed in most cultivars, more leaves were produced in “Wray” and “Roce” in plants of S1 (**Figure 6B**).

**Figure 7:**
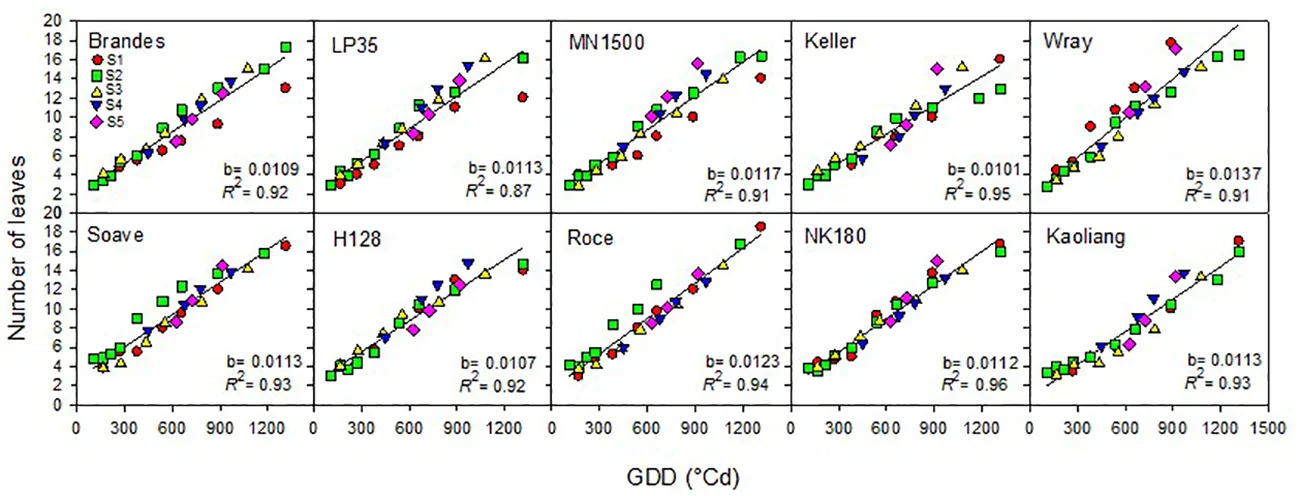
Relationship of the number of leaves per plant vs. GDD in the 10 sorghums at all sowing times (S1: 16 March; S2: 30 March; S3: 16 April; S4: 30 April; S5: 15 May) (year: 2009).

When the number of leaves per plant measured in each cultivar was plotted against thermal time calculated from plant emergence to the emission of the last leaf, close linear regressions (*R*^2^ ≥ 0.87) were found. From a comparison of the *b* coefficients of linear regressions (which indicate the rate of leaf emission per degree day accumulated) of the different cultivars, a faster rate was calculated in “Wray” that, within the same thermal time of the other sorghums, achieved a greater number of leaves. Slow leaf emission rates (*b* ≤ 0.0109) were found in “Brandes”, “Keller”, and “H128” (**Figure 7**).

#### 3.2.5 Dry weight of single plant

**Figure 8:**
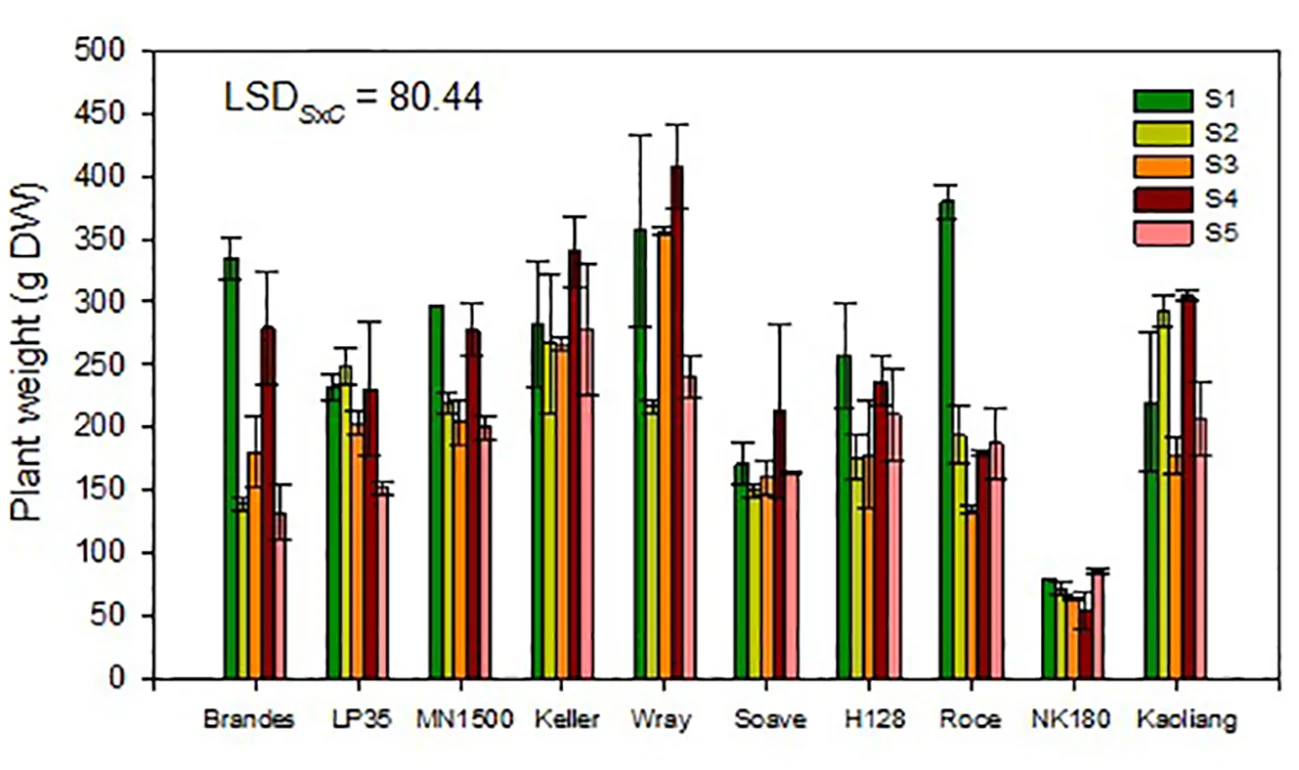
Dry weight of single plant at harvest in sorghum in relation to sowing time (S1: 16 March; S2: 30 March; S3: 16 April; S4: 30 April; S5: 15 May) and cultivar (year: 2009). Small vertical bars indicate the standard error (*n* = 3).

The dry weight of single plant was measured at harvest. Significant effects of both sowing time and cultivar were detected at ANOVA. Across cultivars, bigger plants (>250 g) were produced in S1 and S4. No differences for this trait were observed among the other sowings. Across sowings, the highest weight (>300 g) was measured in “Wray” (sweet type). According to low plant height, small plants (<72 g) were produced in “NK180” (grain type). As reported for the other morphological traits, significant interaction “*S* × *C*” on plant weight was highlighted at ANOVA (*p*< 0.001), and while some cultivars (e.g., “Keller”, “Soave”, and “NK180”) maintained the same plant weight in all sowings, other cultivars (e.g., “Brandes” and “Roce”) produced plants that were bigger when crop was sown very early (S1) (**Figure 8**), as a probable result of less intraspecific competition under lower plant population conditions occurred with mid-March sowing.

**Figure 9:**
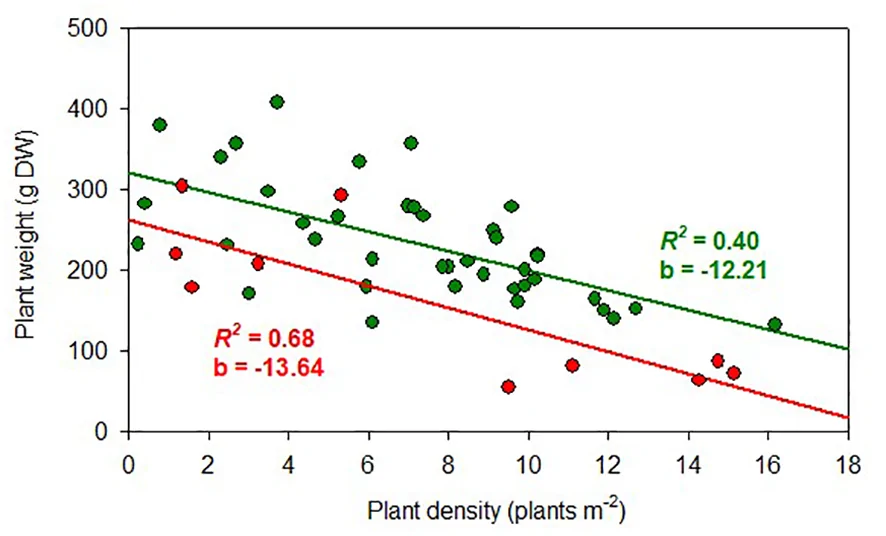
Relationship of plant density at harvest and dry weight of single plant in sorghum (green circles: sweet and fiber types; red circles: grain types). Regression coefficient (*R*^2^) and slope (*b*) are also reported.

Indeed, when the values of plant weight were plotted vs. corresponding plant density, separately for groups of sorghums (that of sweet and fiber types and that of grain types), close relationships were found for both groups, whose negative pattern indicated that single plant weight tended to decrease with the increasing number of plants per unit area (**Figure 9**).

#### 3.2.6 Dry biomass at harvest

**Figure 10:**
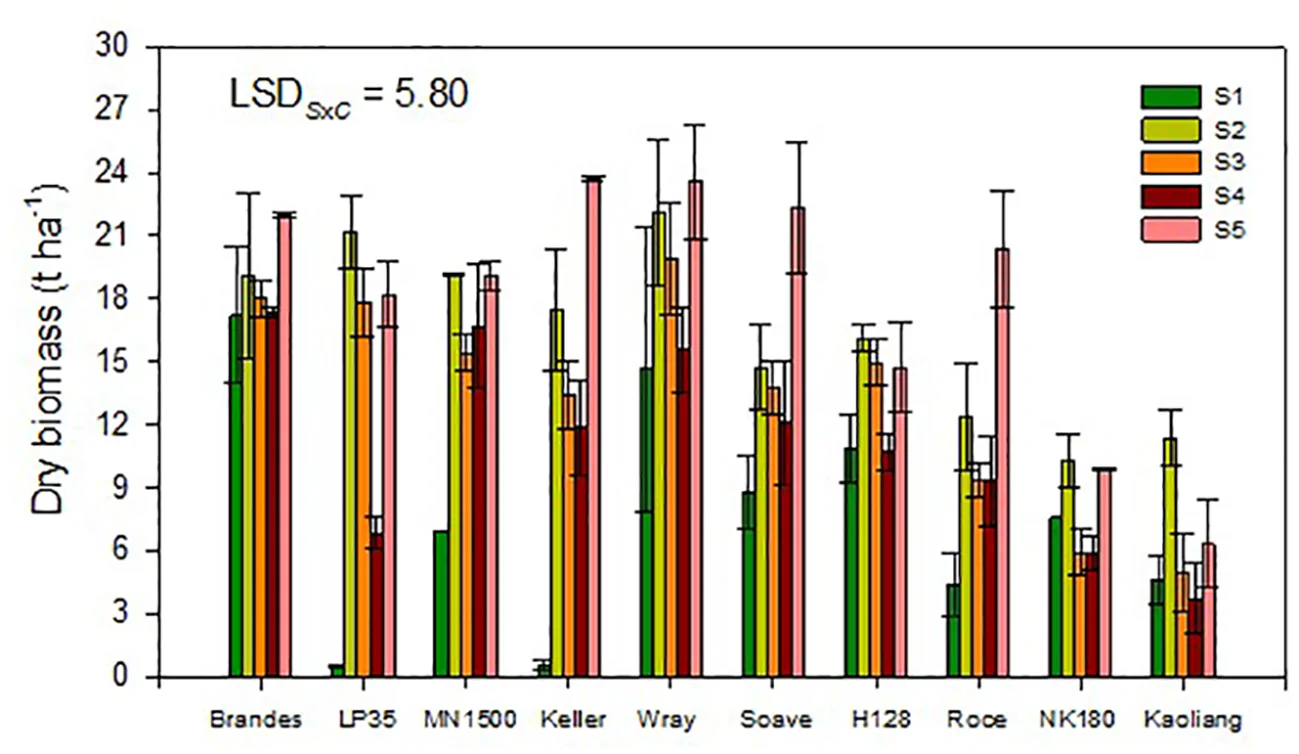
Dry biomass in sorghum in relation to sowing time (S1: 16 March; S2: 30 March; S3: 16 April; S4: 30 April; S5: 15 May) and cultivar (year: 2009). Small vertical bars indicate the standard error (*n* = 3).

Dry biomass measured at harvest was significantly affected by sowing time. Higher biomass was produced by plants sown in late March (S2, 16.39 t ha^−1^), not different from that measured in plants from the latest sowing (S5, mid-May). Very low temperatures experienced during growth by plants of mid-March sowing (S1) decreased plant productivity, which did not reach 10 t ha^−1^. Overall, sweet and fiber types were more productive than grain types. Dry biomass was maximized in “Wray” and “Brandes” (≥19 t ha^−1^). Low biomasses (<8 t ha^−1^) were harvested in “NK180” and “Kaoliang”, both grain types. *Sowing time* and *Cultivar* significantly interacted on this trait (**Figure 10**). Differently from what was observed in some cultivars (e.g., “Brandes”, “Wray”, “H128”, and “NK180”), whose final dry biomass did not change with the time of sowing, in other sorghums (e.g., “LP35”, “MN1500”, “Keller”, “Soave”, and “Roce”), plant productivity was significantly decreased by low temperatures occurring in very early sowing (S1, mid-March), and in some cases (“LP35” and “Keller”), the final biomass did not reach 1 t ha^−1^.

**Figure 11:**
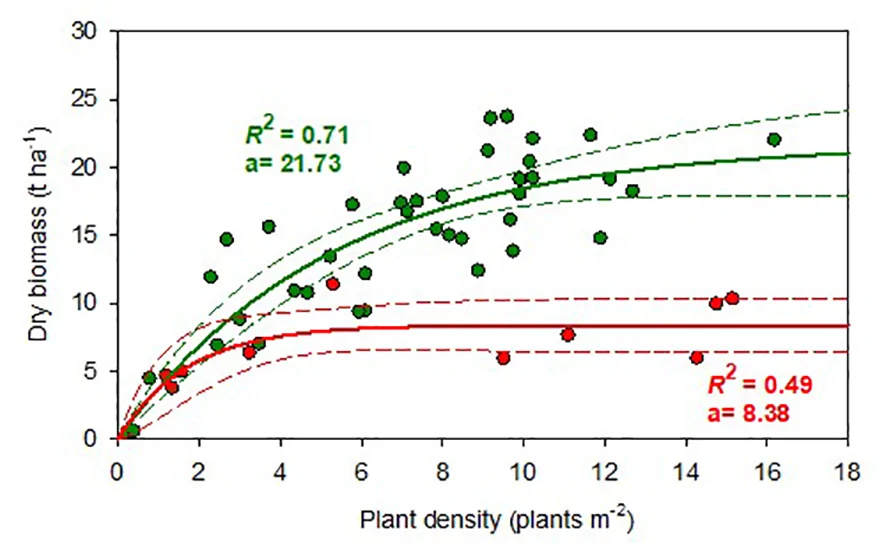
Relationship of plant density at harvest and final dry biomass in sorghum (green circles: sweet and fiber types; red circles: grain types) (short dash lines indicate the 95% confidence intervals).

The relationship between final dry biomass and plant density measured at harvest was studied, separately for the two groups of sorghum: one for the sweet and fiber types (i.e., mostly for biomass production) and one for the grain types (i.e., mostly for grain production). In both cases, the “*exponential rise to a maximum*” course of the relationship, which was closer for the biomass group (*R*^2^ = 0.71), indicated that, in both groups, dry biomass yield tends to increase with the increase in plant density, up to a maximum (*a* = 21.73 and 8.38 t ha^−1^ for biomass and grain sorghums, respectively). After this maximum, the biomass increase becomes null (**Figure 11**).

### 3.3 Field experiment (second year)

#### 3.3.1 First- vs. second-year field emergence

The pool of data of the first- and second**-**year field emergence, limited to the sowings of mid-March (S1) and mid-May (S5), and to cultivars “Brandes” (sweet), “LP35” (sweet), “Keller” (sweet), and “NK180” (grain), was statistically analyzed. Results from a three-way ANOVA indicated significant effects of all the experimental factors (*year*, *sowing time*, and *cultivar*). Field emergence in the second season was slightly but significantly higher than that occurred in the first season (**Table 10**). As expected, sowing in May (S5) determined better performance of seedlings, the emergence of which exceeded 50%. Conversely, across years and cultivars, early sowing in mid-March (S1) led to poor emergence in field (<20%). Among sorghums, “LP35” and “NK180” exhibited overall better seedling emergence (≥40%).

**Table 10:** Effects of year, sowing time, and cultivar on field emergence and final dry biomass in sorghum.

Main effect		Field emergence (%)	Dry biomass (t ha^−1^)
Year (*Y*)	2009	31.37 ± 5.33 b	12.46 ± 1.85 b
	2010	40.83 ± 3.28 a	15.03 ± 1.57 a
Sowing time (*S*)	S1 (16 March)	18.62 ± 2.44 b	8.55 ± 1.35 b
	S5 (15 May)	53.60 ± 2.94 a	18.95 ± 1.37 a
Cultivar (*C*)	Brandes	30.64 ± 5.46 b	20.23 ± 1.16 a
	LP35	39.90 ± 7.04 a	12.14 ± 2.46 b
	Keller	27.54 ± 5.09 b	14.52 ± 3.26 b
	NK180	46.33 ± 6.87 a	8.09 ± 0.51 c
Significance	*Y*	***	**
	*S*	***	***
	*C*	***	***
	*Y* × *S*	***	ns
	*Y* × *C*	ns	*
	*S* × *C*	ns	***
	*Y* × *S* × *C*	ns	ns

For the main effects, values (mean ± SE) followed by the same letter are not significantly different at *p* ≤ 0.05 (Tukey’s test).

Significant at *p* ≤ 0.05 (*), *p* ≤ 0.01 (**), and *p* ≤ 0.001 (***); ns: not significant.

**Figure 12:**
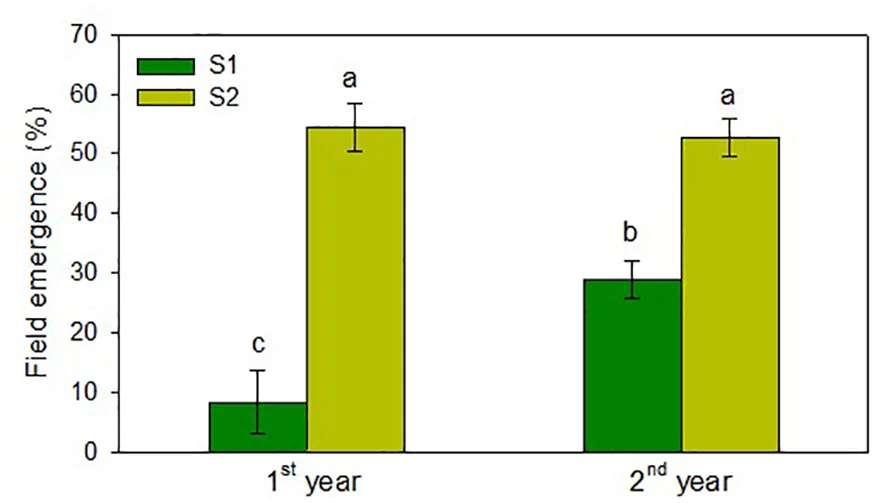
Field emergence in sorghum in the 2 years of experiment in relation to sowing time (S1: mid-March; S5: mid-May). Small vertical bars indicate the standard error (*n* = 12).

Among all the interactions, the only significant one was “*Y* × *S*” (<0.001). In particular, no differences in field emergence between the 2 years were detected under favorable thermal conditions (those experienced by the crop with mid-May sowing), but when sowing was made early in mid-March, significantly more seedlings were counted in the second year (**Figure 12**).

#### 3.3.2 Relationship of plant emergence vs. temperature (first year and second year)

**Figure 13:**
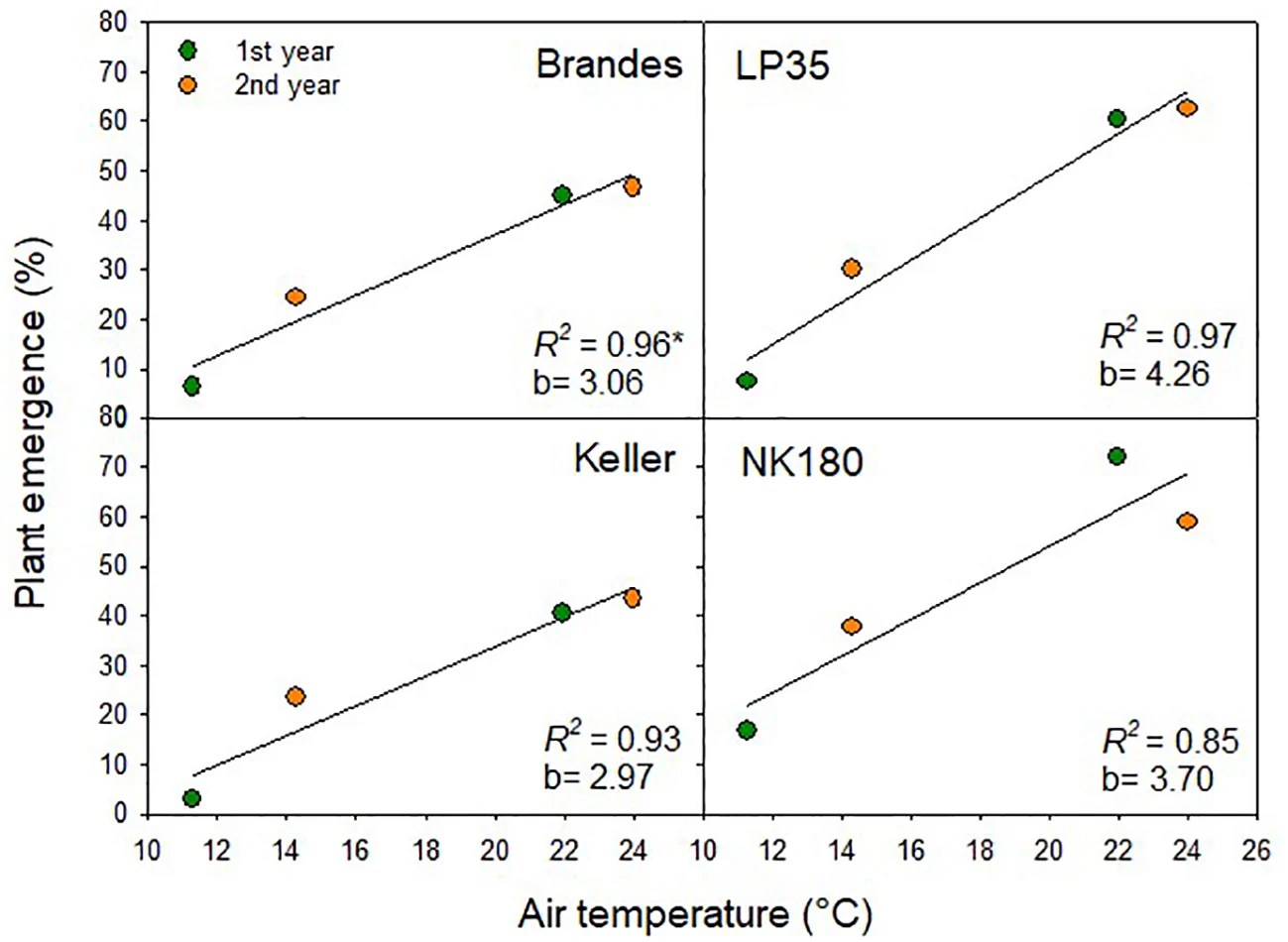
Relationship of plant emergence in field and air *T*_max_ as the average of the 10-day period following sowing, in four cultivars of sorghum.

When data of plant emergence measured in the 2 years of experiment for the two common sowing times (that early in mid-March and that regular in mid-May) were plotted against maximum air temperature as the average of the 10-day period following sowing, strict relationships were found (**Figure 13**). The latter indicate that the extent of plant emergence in the field for the four sorghums depends on the course of maximum air temperatures occurring just after sowing. This result confirms the strict relationships between plant emergence and air temperature, found in the first year in all cultivars except “Kaoliang”.

#### 3.3.3 First- vs. second-year dry biomass

**Figure 14:**
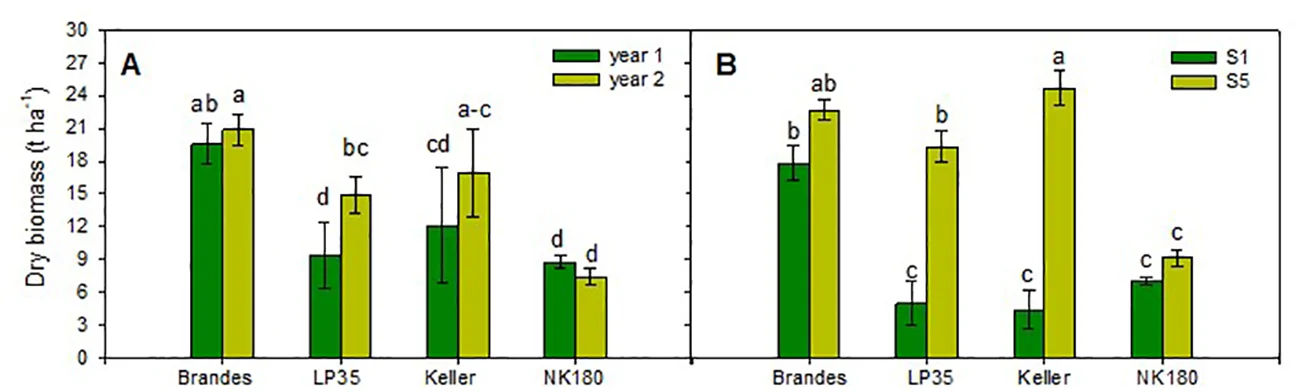
Dry biomass in four cultivars of sorghum in relation to the year of experiment **(A)** and sowing time (**(B)**, S1: 16 March; S5: 15 May). Small vertical bars indicate the standard error (*n* = 6).

Data of final dry biomass measured in the first and second year for sowings of mid-March (S1) and mid-May (S5), and cultivars “Brandes” (sweet), “LP35” (sweet), “Keller” (sweet), and “NK180” (grain), were statistically analyzed altogether at ANOVA. Across sowings and cultivars, a significantly higher biomass (+21%) was obtained in the second season. Across years and cultivars, sowing in mid-May (S5) led to a greater biomass (18.95 t ha^−1^). Among cultivars, the most yielding was “Brandes”, which produced an average biomass of 20.23 t ha^−1^. As expected, low biomass (8.09 t ha^−1^) was produced in “NK180” (grain type). Significant interaction “*Y* × *C*” (*p*< 0.001) was found at ANOVA. Indeed, differently from the other sorghums whose final biomass did not change with the year of experiment, “LP35” provided a significantly higher biomass in the second year (**Figure 14A**). “Cultivar” also significantly interacted with “sowing time” (“*S* × *C*”, *p*< 0.001), and while the productivity of “Brandes” and “NK180” was not affected by the time of sowing, much higher biomasses were provided by “LP35” and “Keller” when sowing was postponed from mid-March to mid-May (**Figure 14B**).

## 4 Discussion

Being native to tropical areas (Tari et al., 2013), sorghum requires warm temperatures to grow, and chilling temperatures strongly limit its geographical distribution. Cool temperatures may depress seed germination and seedling emergence, which are considered as the most sensitive stages of the crop growth (Casto et al., 2021).

In agreement with previous studies, our results showed that temperatures below 10 °C markedly delayed and reduced germination, confirming that in early spring sowings in semi-arid regions, sorghum may face significant establishment constraints (Ercoli et al., 2004). Although sorghum is generally considered drought-resistant, prolonged dry periods associated with climate change may compromise its performance (Eggen et al., 2019). Therefore, identifying cultivars capable of germinating and emerging under low-temperature open-field conditions and adapting to early sowing is essential in semi-arid regions. This strategy allows crops to escape the dry season and achieve significant savings in irrigation water, which is a crucial objective under current global warming scenarios.

Results from this study revealed that poor seedling emergence occurred in very early sowing (mid-March). Low temperatures can cause injuries in plant embryos of germinating seeds (Salas Fernandez et al., 2014). However, a genetic variation was observed in the crop response to low temperatures during seed germination and seedling emergence. Seed germination percentage >60% under a controlled temperature of 8 °C in the laboratory were obtained in some cultivars (“H128”, fiber type, “Wray”, sweet type, and “Kaoliang”, grain type). In this regard, these sorghums may represent good candidates for early sowings in drylands of Southern Europe. Previous studies on seed germination of sorghum under controlled conditions confirmed the existence of a genetic variation in the germination response to low temperatures, suggesting the possibility to screen for high tolerance to cold during germination and following seedling establishment in the field within the existing germplasm of sorghum (Yu et al., 2004; Patanè et al., 2021). Some authors proposed “low base temperature” and “low thermal time requirements” for germination as criteria for the selection of cultivars adapted to early spring sowings for the semi-arid regions of Southern Europe. These traits should be combined with adequate levels of biomass productivity (Patanè et al., 2021).

However, in this study, when the experiment was moved from laboratory to open-field conditions, different results were observed. Indeed, an overall poor emergence occurred with the earliest planting (that in mid-March). In this sowing, seeds were exposed to very low temperatures (<10 °C) at the time of sowing, which strongly decreased seedling emergence (<15%), even in those cultivars that performed well in laboratory experiments for germination assessment at 8 °C. This suggests that poor emergence with mid-March planting is the result of a combined inhibitory effect of very cool temperatures, which are the prevailing determining factor, and unfavorable soil conditions (e.g., planting depth and oxygen availability) upon seed germination and seedling establishment (Evert et al., 2009).

Therefore, seed germination in laboratory tests may not be considered a reliable predictor of the extent of field emergence of sorghum under restricted environmental conditions, when the latter are too constrained. Salas Fernandez et al. (2014) also found no relationship between germination under a controlled environment and field emergence under cold temperatures. Nevertheless, laboratory tests may provide information on the potential behavior of the different cultivars in the field when early sowing is adopted. This assumption was also confirmed by the strict positive relationship (*r* = 0.94**) found between final germination percentage in laboratory at 8 °C and plant emergence in the field in mid-March sowing. Significant correlations were reported in literature between field emergence and germination as assessed in the laboratory, in sorghum as well (Brar and Stewart, 1994; Tiryaki and Andrews, 2002), but some inconsistencies in field emergence prediction were also highlighted (Kolasinska et al., 2000).

When the final field emergence was regressed vs. maximum air temperatures, as the average of the 10-day period after sowing, the value of the *x*-axis intercept of this relationship was assumed as an estimation of the theoretical minimum or base thermal threshold for plant emergence. A base thermal threshold calculated in cultivar “Kaoliang” much lower than that of the other sorghums confirms the great cold tolerance of this Chinese group of sorghums (Casto et al., 2021). These sorghums can represent a good source of genetic diversity for sorghum breeding; however, they present unfavorable agronomic traits such as low biomass productivity, as revealed in this study.

Although a strict relationship was also found between plant emergence in the field and plant population at harvest, in some cultivars (e.g., “Brandes”), a final plant density higher than that of the other sorghums (except “NK180”) was measured at harvest, in plots of the earliest sowing (that of mid-March), although seedling emergence was rather poor. A reasonable explanation for this result is the occurrence of a possible thermo-dormancy in this cultivar, which lengthened but did not prevent seed germination at very low temperatures, as an adaptive mechanism to survive very stressful thermal conditions (Harris et al., 1987). In this way, the crop recovered from the cold and new seedlings probably emerged later, when the seedling emergence score was already stopped. Indeed, seeds of “Brandes” were also slow in germination (MGT) as assessed via laboratory testing at 8 °C, and an overall strict relationship (*r* = 0.97***) of field emergence vs. germination speed (MGT), even closer than that vs. final germination percentage, was also described. Previous studies on seed germination of sorghum conducted under controlled temperatures highlighted the existence of cultivars with low thermal threshold (base temperature) for germination, but slow in germination, thus requiring a longer thermal time to complete germination (Patanè et al., 2021). A close relationship between field emergence and the speed of seed germination in the laboratory were also reported in literature for sorghum (Yousif, 2010). Thermo-dormancy probably did not occur in other cultivars (e.g., “LP35”) that had not only poor seedling emergence like “Brandes” but also low final plant population, in the earliest sowing.

Among cultivars, only “NK180” achieved the predicted plant density (11 plants m^−2^) at the earliest sowing (mid-March). In this regard, this sorghum may be suitable for early planting in semi-arid areas. Other cultivars (e.g., “H128”), having a field emergence similar to that of “NK180” in this sowing, exhibited a plant population that was <4.5 plants m^−2^. We may assume that, in these cultivars, a high rate of plants did not survive the cold temperatures.

Fewer leaves per plant and lower plant height were measured in plants of sorghum at the two sowings in March. Previous studies on sorghum reported reduced leaf number and plant height, besides a low seed germination rate, at temperatures from 8 °C to 10 °C (Major et al., 1982). An increase in membrane rigidity and an alteration in membrane-localized proteins, which promote the accumulation of reactive oxygen species (ROS) (Liu et al., 2018), with further damages upon plant photosystems, have been suggested as responsible for the detrimental effects of low temperatures upon plant growth and leaf emission in sorghum (Casto et al., 2021). However, chilling did not always inhibit plant growth, as revealed by final plant height in which some cultivars (“Brandes”, “Soave”, “NK180”, and “Kaoliang”) did not change with sowing time. These results demonstrate that minimum temperatures for growth are lower for certain cultivars. Greater contribution of leaves on total plant biomass partitioning involves an overall higher ash content in plants of energy crops, affecting combustion (Monti et al., 2008). Indeed, plant biomasses low in ash content are preferable as solid biofuel for thermal utilization (Morales et al., 2024).

In this study, biomass yield was not consistent across sowing times, but fluctuated depending on the cultivar. It was overall low in the earliest sowing (mid-March), but when sowing was shifted to late March, in some cultivars, biomass yield matched (“LP35” and “MN1500”) or was even higher (“Kaoliang”) than that measured in mid-May sowing (control), and 27% of irrigation water was saved. In addition, cultivars cold tolerant during germination and seedling establishment in the field did not rank among the best-performing cultivars in final biomass yield. Indeed, yield potential is a genetic trait (Habyarimana et al., 2020), and cultivars like “NK180” (dual-purpose sorghum, for feed and biomass), despite performing well in the earliest sowings, produced a final dry biomass similar to or even lower than that of most biomass types in the same sowings. Among biomass sorghums, “H128” (fiber type) in the earliest sowing gave a final dry biomass close to that obtained in mid-May sowing. However, as an early-maturity cultivar, this sorghum maintained an overall lower productivity than that of late-maturity types. On the other hand, earlier cultivars could be preferred over long-season ones (different from most biomass types in this study, “H128” achieved the maturity stage). In fact, ripening 1 or 2 weeks earlier in the cultivation areas of Southern Europe can reduce plant moisture content by up to 20%–30% (Pannacci and Bartolini, 2016). Lower moisture content in biomass crops reduces energy and costs associated with drying (Junpeng et al., 2020).

Among sweet types, the cultivar “Brandes” in the earliest sowing produced a biomass as high as in the last sowing (mid-May), although with lower field emergence and lower plant population. In this cultivar, less plant competition under high plant spacing (low plant density) in mid-March sowing led to plants that developed taller and heavier stems, thus compensating for the low plant density. Similar behavior was observed in the fiber type “H128”. This result can be considered as a crop strategy to maintain adequate levels of biomass yield under low plant population (Berenguer and Faci, 2001). Thick-stemmed plants are reported as preferable to thin-stemmed plants in sorghum for energy biomass production, because thick stems contribute to higher biomass and are less prone to lodging than thin stems (Kong et al., 2020). Long-season, tall and thick stalk sweet sorghum cultivars are also reported as desirable traits for production of highly recoverable concentrated sugars in sweet sorghums (Regassa and Wortmann, 2014). High and heavy plants greatly contributed to the high levels of biomass produced in the cultivar “Wray” of sweet sorghum at all sowings, despite the poor rate of field emergence. Great productivity levels (>27 t ha^−1^) were reported in literature for this cultivar (Dolciotti et al., 1998). As a multi-purpose sweet sorghum (for both ethanol and energy biomass production), “Wray” can be considered as a good option for cultivation areas of Southern Europe. Other sorghums (“Keller” and “LP35”, both sweet types) were quite productive, even with early sowing in late March, but when they experienced very cool conditions (those of the earliest sowing in mid-March), their productivity was noticeably decreased (<1 t ha^−1^); therefore, these cultivars are not resilient to cold.

Data of the second year of experiment, although limited to two sowing times and four cultivars, confirmed the results obtained in the first season. Sowing in mid-March was too early for most of the cultivars assessed, at least in the site where the experiment was conducted (500 m a.s.l.). In this respect, early-season studies on sorghum to be conducted in warmer sites (at lower altitude) are suggested. Nevertheless, it is important to highlight that in both years, the cultivar “Brandes” maintained stable levels of final biomass, even when sown in mid-March.

However, several challenges remain that limit a full understanding of the genetic basis of cold tolerance in sorghum, to be introduced in breeding programs of this species for better adaptation to early sowings.

## 5 Conclusions

This study highlighted the existence of cultivars possessing cold tolerance during germination and subsequent seedling emergence in the field within the available germplasm of sorghum, suitable to early sowings in the semi-arid areas of Southern Europe. High resilience to cold in the field was found in sorghum “NK180” that, although low-yielding even in regular sowings, may represent a valuable genetic source for breeding programs to enhance chilling tolerance in the field during the very early stages of growth, among the existing cultivars of sorghum, or to develop new cultivars that combine cold resilience and high productivity. The cultivar “H128”, also resistant to cold, showed more stable production over sowing times, thus becoming agronomically more suitable (**Supplementary Table 1**). Great yield stability was also found in the cultivar “Brandes”, which provided high plant stands and great final biomasses, even under sowing in early March, despite only moderate cold tolerance.

An alternative agronomic approach to improving yields when extremely low temperatures are expected to occur in mid-March is to slightly delay sowings, in this case, using more productive cultivars of sorghum. Indeed, the results from this study indicated that even a 15-day shift of sowing from mid-March to early April led to biomass yields >20 t ha^−1^ in some cultivars, even in those (“LP35”, “Keller”, and “Wray”) less tolerant to cold, saving the same amount of irrigation water of sowing in early March.

Although seed germination in the laboratory may not be considered a reliable predictor of successful field emergence under very limiting environmental conditions, it can help in a first screening within the available germplasm of sorghum for cold tolerance.

## Data Availability

The raw data supporting the conclusions of this article will be made available by the authors, without undue reservation.
