# Supplementary material for: Assessment of cold tolerance in early field emergence in sweet, fiber, and grain sorghums [Sorghum bicolor (L.) Moench]
Source: Front Plant Sci. 2026 Sep 9;17:1842879. doi: 10.3389/fpls.2026.1842879 (PMC13597681; doi:10.3389/fpls.2026.1842879)
Supplement: Supplementary file 1 [file DataSheet1.pdf]

Supplementary Table 1

Summary table.

Cultivars of sorghum in laboratory experiment	Brandes, LP35, MN1500, Keller, Wray, H128, Kaoliang
Cultivars of sorghum (first year open-field experiment)	Brandes, LP35, MN1500, Keller, Wray, Soave, H128, Roce, NK180, Kaoliang
Cultivars of sorghum (second year open-field experiment)	Brandes, LP35, Keller, NK180
Sowing dates (first year open-field experiment)	March 16 (S1), March 30 (S2), April 16 (S3), April 30 (S4), May 15 (S5)
Sowing dates (second year open-field experiment)	March 16 (S1), May 15 (S5)
First-year air temperatures in field (average Tmin and average Tmax of the 10-day period following sowing)	4.2-11.3°C (S1); 10.5-19.6°C (S2); 8.4-16.7°C (S3); 10.2-17.4°C (S4); 11.7-21.9°C (S5)
Second-year air temperatures in field (average Tmin and average Tmax of the 10-day period following sowing)	6.5-15.3°C (S1); 14.0-24.6°C (S5)
Best cold tolerant cultivars	NK180, H128
